# Piceatannol Protects PC-12 Cells against Oxidative Damage and Mitochondrial Dysfunction by Inhibiting Autophagy via SIRT3 Pathway

**DOI:** 10.3390/nu15132973

**Published:** 2023-06-30

**Authors:** Jie Liu, Peishi Mai, Zihui Yang, Zongwei Wang, Wei Yang, Ziyuan Wang

**Affiliations:** 1 Key Laboratory of Geriatric Nutrition and Health, Ministry of Education, Beijing Technology and Business University (BTBU), Beijing 100048, China; 2 China-Canada Joint Lab of Food Nutrition and Health (Beijing), Beijing Technology and Business University (BTBU), Beijing 100048, China; 3 College of Basic Science, Tianjin Agricultural University, Tianjin 300392, China

**Keywords:** piceatannol, oxidative damage, mitochondrial function, autophagy, SIRT3

## Abstract

Oxidative stress has been identified as a major cause of cellular injury in a variety of neurodegenerative disorders. This study aimed to investigate the cytoprotective effects of piceatannol on hydrogen peroxide (H_2_O_2_)-induced pheochromocytoma-12 (PC-12) cell damage and explore the underlying mechanisms. Our findings indicated that piceatannol pre-treatment significantly attenuated H_2_O_2_-induced PC-12 cell death. Furthermore, piceatannol effectively improved mitochondrial content and mitochondrial function, including enhancing mitochondrial reactive oxygen species (ROS) elimination capacity and increasing mitochondrial transcription factor (TFAM), peroxisome-proliferator-activated receptor-γ coactivator-1α (PGC-1α) and mitochondria Complex IV expression. Meanwhile, piceatannol treatment inhibited mitochondria-mediated autophagy as demonstrated by restoring mitochondrial membrane potential, reducing autophagosome formation and light chain 3B II/I (LC3B II/I) and autophagy-related protein 5 (ATG5) expression level. The protein expression level of SIRT3 was significantly increased by piceatannol in a concentration-dependent manner. However, the cytoprotective effect of piceatannol was dramatically abolished by sirtuin 3 (SIRT3) inhibitor, 3-(1H-1,2,3-Triazol-4-yl) pyridine (3-TYP), which led to an exacerbated mitochondrial dysfunction and autophagy in PC-12 cells under oxidative stress. In addition, the autophagy activator (rapamycin) abrogated the protective effects of piceatannol on PC-12 cell death. These findings demonstrated that piceatannol could alleviate PC-12 cell oxidative damage and mitochondrial dysfunction by inhibiting autophagy via the SIRT3 pathway.

## 1. Introduction

Oxidative stress, a hallmark of aging, acts as the main risk factor for most neurodegenerative diseases including Alzheimer’s disease (AD) and Parkinson’s disease (PD) [1,2]. Reactive oxygen species (ROS) can be generated from the impairment of metabolic function under oxidative stress [3]. Excessive ROS from oxidative stress led to mitochondrial dysfunction, causing neuron injury [4]. The mitochondrion is essential for the maintenance of cellular homeostasis involved in cell processes relevant to neuronal function, including autophagy [5]. Cai et al. found that mitochondrial ROS generation could decrease mitochondrial membrane potential (MMP), activating cell autophagy [6]. Moreover, ROS participates as a signal molecule associated with autophagy, considered as the main mechanism of oxidative stress-caused neurodegenerative disorders [7]. Autophagy is a highly conserved cellular process of the degradation of intracellular organelles and components by lysosomes [8]. The autophagic pathway plays an important role in the differentiation, growth, maintenance and homeostasis of neuronal cells. Glycine was demonstrated to attenuate oxidative injury in neurons via reducing autophagy both in vitro and in vivo [6]. Meanwhile, the dysregulation of autophagy has been shown to contribute to the pathogenesis of neurological diseases [9]. Thus, the regulation of autophagy could be a potential strategy to prevent neurological disease processes.

Sirtuin 3 (SIRT3), a nicotinamide adenine dinucleotide (NAD^+^)-dependent enzyme, is highly expressed in the nervous system and vital to physiological neuronal processes and brain functions, which modulates mitochondrial function, metabolism and oxidation responses [10,11]. Shulyakova et al. showed that SIRT3 overexpression protected differentiated pheochromocytoma-12 (PC-12) cells against H_2_O_2_-induced cell death [12]. Moreover, SIRT3 plays an important role in autophagy [13]. SIRT3 exhibits inhibitory or promotive effects on hepatic cell autophagy through different mechanisms in distinct physiological contexts [14]. Similarly, in neuronal cells, the regulatory role of SIRT3 on the autophagy process is different in different cell models [15,16]. 

Several clinical studies have implicated neuroprotective diets enriched with plant polyphenols, which promoted memory, cognition, and other brain functions via suppressing oxidative stress and neuroinflammation from neurotoxin damage [17]. Piceatannol (3,3’,4’,5-tetrahydroxy-trans-stilbene), a naturally occurring stilbene, has been evidenced with many bioactivities, such as antioxidant, anti-inflammation, anti-diabetes, anti-cancer, cardio-protection and neuroprotection [18,19,20,21,22,23,24,25]. Previous studies showed that piceatannol displayed effective cytoprotection of nerve cells against H_2_O_2_-induced oxidative stress [22]. However, the underlying neuroprotection mechanism for the therapeutic role of piceatannol needs to be fully elucidated. We previously found that piceatannol attenuated oxidative damage and mitochondrial-mediated apoptosis, exerting a cytoprotective effect in PC-12 cells through SIRT3/Forkhead box O3 (FOXO3a) signaling pathway [23]. Meanwhile, piceatannol improved the cognitive function of AD mice by reducing oxidative stress, neuroinflammation and beta-amyloid (Aβ) production in the hippocampus [24]. However, whether piceatannol plays the cytoprotective effects through SIRT3-mediated autophagy in PC-12 cells needs to be further clarified.

In the present study, the neuroprotective effects of piceatannol against H_2_O_2_-induced mitochondrial dysfunction and autophagy overactivation in PC-12 cells were investigated. 3-(1H-1,2,3-Triazol-4-yl) pyridine (3-TYP), SIRT3 inhibitor, and rapamycin (autophagy activator) were used to explore the underlying mechanism of piceatannol-treated PC-12 cells viability under oxidative stress. The current research could provide new insights into the prevention and management of neurodegenerative diseases by dietary supplementation of piceatannol.

## 2. Materials and Methods

### 2.1. Materials and Reagents

Piceatannol, dimethyl sulfoxide (DMSO), and bovine serum albumin (BSA) were obtained from Sigma-Aldrich (St. Louis, MO, USA). RPMI 1640 medium, horse serum (HS), fetal bovine serum (FBS), Hank’s balanced salt solution (HBSS) and penicillin/streptomycin solution were purchased from Gibco (Grand Island, NY, USA). 3-TYP and Cell-Counting-Kit-8 (CCK8) were purchased from Med Chem Express (Monmouth Junction, NJ, USA). MitoSOX™ Red, SYTO™ 9 and MitoTracker™ Red CMXRos were obtained from Invitrogen (Grand Island, NY, USA). Bicinchoninic acid (BCA) protein assay kits were purchased from Thermo Fisher Scientific (Waltham, MA, USA). JC-1 Mitochondrial Membrane Potential Assay Kit was purchased from Abcam (Cambridge, UK). Autophagy Staining Assay Kits with monodansylcadavrine (MDC) and propidium iodide (PI) were obtained from Beyotime Biotechnology (Shanghai, China). Antibodies against mitochondrial transcription factor (TFAM) (A3173), Complex IV (A17889), peroxisome-proliferator-activated receptor-γ coactivator-1α (PGC-1α) (A20995), light chain 3B (LC3B) (A19665), autophagy-related protein 5 (ATG5) (A19677) and goat anti-rabbit IgG H&L (HRP) (AS014) were obtained from ABclonal Technology Co., Ltd. (Wuhan, China).

### 2.2. Cell Culture

Rat pheochromocytoma cells (PC-12 cells) were purchased from American Type Culture Collection (ATCC, Manassas, MD, USA). PC-12 cells were cultured in RPMI 1640 medium supplemented with 10% HS, 5% FBS, 100 U/mL penicillin and 100 μg/mL streptomycin and incubated in a humidified atmosphere at 37 °C containing 5% CO_2_.

### 2.3. Cell Viability

PC-12 cells were seeded in a flat-bottom 96-well plate at a density of 2 × 10^4^ cells/well for 24 h. PC-12 cells were incubated with different concentrations of piceatannol for 24 h and induced by H_2_O_2_ for another 24 h. Then, cell viability was investigated using CCK8 assay which was a sensitive colorimetric assay for the determination of cell viability. CCK8 solution (10 μL) was added to each well and incubated with PC-12 cells at 37 °C for 1 h according to the manufacturer’s protocol. Subsequently, the cell viability was detected by a microplate reader (Spark, Tecan, Switzerland) at 450 nm wavelength.

### 2.4. Measurement of Mitochondrial Superoxide Level

The accumulation of mitochondrial superoxide was detected using MitoTracker™ Red CMXRos according to the manufacturer’s protocol. PC-12 cells were seeded in 12-well plates at a density of 2 × 10^5^ cells/well for 24 h. After treatments, the cells were washed by PBS and then MitoSOX™ reagent was used to stain the mitochondrial ROS for 20 min in the dark. The nucleic acids were stained with SYTO™ 9 reagent for another 2 min at 37 °C. After incubation, the fluorescence intensities were measured using a microplate reader (Spark, Tecan, Switzerland) with Excitation/Emission wavelength at 581/644 nm for MitoSOX™ and 485/498 nm for SYTO™ 9. The fluorescence image was observed under a fluorescence microscope (IX71, Olympus, Japan).

### 2.5. Measurements of Mitochondrial Membrane Potential (MMP)

The MMP of PC-12 cells was detected using JC-1 Mitochondrial Membrane Potential Assay Kit. PC-12 cells were placed in 12-well plates for 24 h and treated with piceatannol for 24 h, followed by incubating with 250 μM H_2_O_2_ for another 24 h. Then, 1000 μL JC-1 dye loading solution was pipetted to each well and incubated with PC-12 cells for 20 min at 37 °C. Subsequently, the MMP was determined at a concentration of 2 × 10^5^ cells/mL using a flow cytometry (CytoFLEX S, Beckman, CA, USA) and analyzed by CytExpert software 2.4.0.28 (Beckman, CA, USA).

### 2.6. Detection of Autophagy

The autophagy of PC-12 cells was detected using Autophagy Staining Assay Kit (Beyotime, Shanghai, China) according to the manufacturer’s protocol. Briefly, PC-12 cells were pretreated with piceatannol for 24 h followed by incubating with 250 μM H_2_O_2_ for 24 h. Then, PC-12 cells were stained with the MDC and PI dye-loading solution for another 1 h. After staining, the fluorescence of PC-12 cells at a concentration of 2 × 10^5^ cells/mL was detected using flow cytometry (CytoFLEX S, Beckman, CA, USA) and analyzed by CytExpert software (Beckman, CA, USA).

### 2.7. Western Blotting

PC-12 cells (2 × 10^5^ cells/mL) were seeded in 12-well plates for 24 h. After treatment with piceatannol for 24 h, cells were treated with or without 3-TYP or rapamycin for 4 h, followed by H_2_O_2_ treatment for another 24 h, PC-12 cells were lysed and collected with radio-immunoprecipitation assay buffer (RIPA) mixed with phosphatase inhibitor and protease inhibitor cocktail (Sigma Aldrich, St. Louis, MO, USA). Whole-cell protein extracts were measured by BCA protein assay kits (Thermo Fisher Scientific, Waltham, MA, USA) and denatured at 95 °C for 5 min. Subsequently, 40 μg protein of each sample was uploaded and fractionated by a 12% SDS-PAGE gel and then transferred to an NC membrane (Pall Life Sciences, Port Washington, NY, USA). After washing with Tris-buffered saline containing 0.1% Tween-20 (0.1% TBST), the NC membrane was blocked with 5% (*w*/*w*) skim milk in TBST for 1 h. Then the membrane was incubated with primary antibodies (1:1000) at 4 °C overnight and secondary antibodies at 37 °C for 2 h. Protein bands were visualized and analyzed using a Bio-Rad imaging system (Bio-Rad Laboratories, Hercules, CA, USA).

### 2.8. Statistical Analysis

The data in this study were expressed as the mean ± standard deviation (SD). Statistical analyses were carried out by one-way ANOVA using SPSS V. 26 (SPSS, Chicago, IL, USA). *p* < 0.05 was considered to demonstrate statistically significant differences.

## 3. Results

### 3.1. Piceatannol Attenuated PC-12 Cells Mitochondrial Dysfunction Induced by H_2_O_2_

**Figure 1:**
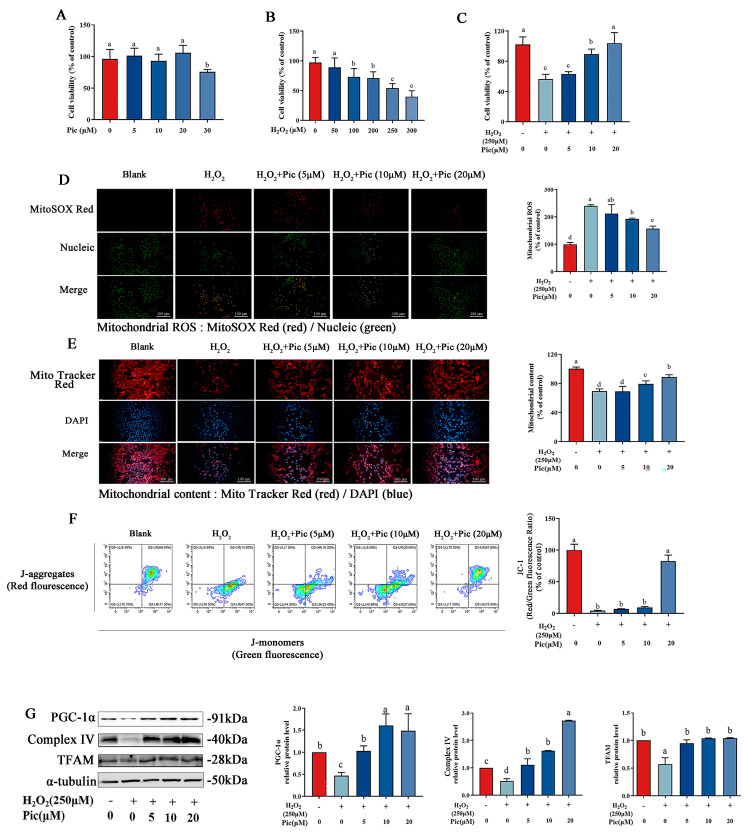
Piceatannol attenuated mitochondrial dysfunction of PC-12 cells under oxidative stress. (**A**) Cell viability of PC-12 cells treated with different concentrations of piceatannol (Pic) (0–30 μM). (**B**) Cell viability of PC-12 cells treated with different concentrations of H_2_O_2_ (0–300 μM). (**C**) Cell viability of PC-12 cells preincubated with piceatannol (0, 5, 10 and 20 μM) for 24 h, followed by incubation with or without 250 μM H_2_O_2_ for another 24 h. Mitochondrial ROS (**D**) and mitochondrial content (**E**) observed by fluorescence microscope (Magnification, ×100; Scale bar = 100 μm). (**F**) Mitochondrial membrane potential measured by flow cytometer. (**G**) The protein expression level of PGC-1α, Complex IV and TFAM measured by Western blotting assay. The data for the experiments were represented as the means ± standard deviation (SD). Results marked with the same letters were not significantly different (*p* < 0.05).

To determine the toxicity effect of H_2_O_2_ and piceatannol on PC-12 cells, cell viability was evaluated after 24 h exposure to different concentrations of H_2_O_2_ or piceatannol by using CCK8 assay. As shown in Figure 1A, piceatannol showed no cytotoxic effect on PC-12 cells at concentrations up to 20 μM (*p* > 0.05). However, the cell viability significantly decreased following incubation with piceatannol at a concentration over 30 μM. The cell viability of PC-12 dramatically decreased in a concentration-dependent manner after H_2_O_2_ treatment (50–300 μM) (Figure 1B). In addition, H_2_O_2_ treatment (250 μM) almost led to 50% cell death, which was used for the subsequent experiments to evaluate the protective effect of piceatannol. To further explore the protective effect of piceatannol on H_2_O_2_-induced PC-12 cell oxidative damage, PC-12 cells were incubated with piceatannol (5–20 μM), followed by exposure with 250 μM H_2_O_2_ for another 24 h. The results revealed that piceatannol pretreatment significantly increased cell viability in a concentration-dependent manner, compared to cells induced by H_2_O_2_ alone. Pre-incubation with 5, 10 and 20 μM of piceatannol prevented cell death under oxidative stress to 63%, 90% and 104%, respectively (Figure 1C). Moreover, H_2_O_2_ treatment significantly upregulated mitochondrial ROS fluorescent signal in PC-12 cells compared to the control group, while pretreatment with piceatannol significantly lowered mitochondrial ROS production (Figure 1D). According to MitoTracker Red CMXRos staining result, the piceatannol supplement group gained a higher level of red fluorescence than cells treated with H_2_O_2_ alone, indicating that piceatannol attenuated mitochondrial content loss (Figure 1E). Furthermore, the flow cytometry results showed that H_2_O_2_ notably decreased the red/green fluorescence ratio in PC-12 cells, indicating impaired mitochondrial integrity. Whereas, pretreatment with piceatannol dramatically promoted the red/green fluorescence ratio in PC-12 cells in a concentration-dependent manner (Figure 1F). Results of Western blot revealed that H_2_O_2_ dramatically decreased the protein expression of PGC-1α, Complex IV and TFAM in PC-12 cells, which was upregulated by piceatannol pretreatment (Figure 1G). These results illustrated that piceatannol could ameliorate the mitochondrial dysfunction of PC-12 cells under oxidative stress. 

### 3.2. Piceatannol Decreased H_2_O_2_-Induced Autophagy in PC-12 Cells

**Figure 2:**
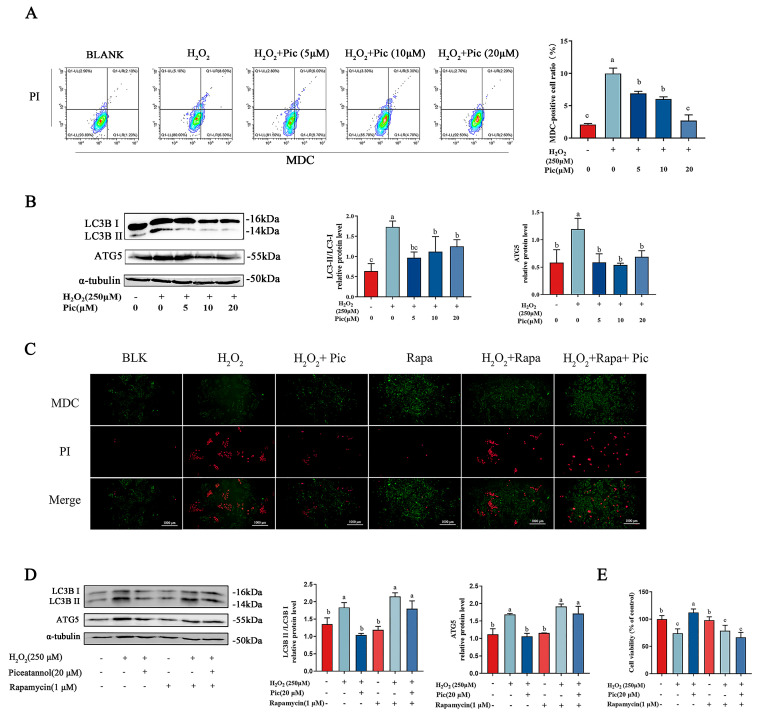
Piceatannol attenuated PC-12 cell death and autophagy induced by H_2_O_2_. (**A**) PC-12 cells were pretreated with piceatannol for 24 h, exposed to 250 μM H_2_O_2_ for another 24 h. Then cells were stained with MDC and PI fluorescence probe and detected by flow cytometer. (**B**) The protein expression level of LC3B II/I and ATG5 measured by Western blotting. PC-12 cells were incubated with piceatannol for 24 h, then treated with rapamycin (Rapa) for 4 h and H_2_O_2_ for another 24 h. (**C**) The MDC/PI probe staining observed by fluorescence microscope (Magnification, ×100; Scale bar = 100 μm). (**D**) The protein expression of LC3B II/I and ATG5 measured by Western blotting assay. (**E**) The cell viability detected by CCK8 assay. The data for the experiments were represented as the means ± standard deviation (SD). Results marked with the same letters were not significantly different (*p* < 0.05).

Autophagy plays a prominent role in cellular damage and cell death [26,27]. The autophagy of PC-12 cells was determined by using MDC, a specific acidic vesicular organelles (AVOs) fluorescence staining pigment, which was often used to observe intracellular autophagosomes. Moreover, PI staining was used to determine the cellular death of PC-12. As shown in Figure 2A, the flow cytometry result indicated an elevated percentage of PC-12 cells with MDC and PI signaling after exposure to H_2_O_2_, while pre-incubation with piceatannol significantly reduced the ratio of positive MDC and PI signaling. These results suggested that piceatannol markedly inhibited the overactive autophagy of PC-12 cells. Furthermore, compared to the control group, the expression level of LC3B II/I and ATG5 were notably upregulated in PC-12 cells induced by H_2_O_2_. While the expression level of these proteins was markedly downregulated in piceatannol-pretreated groups, compared to H_2_O_2_ treatment alone (Figure 2B). 

To investigate the mechanism of the inhibitory effect of piceatannol, an autophagy activator rapamycin was applied in PC-12 cells cultured with H_2_O_2_. According to MDC/PI fluorescent intensity, rapamycin significantly enhanced the autophagosome formation which was reduced by piceatannol (Figure 2C). Western blotting results revealed that piceatannol pre-incubation significantly down-regulated the ratios of LC3B-II/I and reduced the expression of ATG5 in H_2_O_2_-induced PC-12 cells, while rapamycin significantly promoted the expression of LC3B-II/I and ATG5 even after piceatannol pretreatment (Figure 2D). These findings suggested that rapamycin alleviated autophagic inhibition by piceatannol in PC-12 cells under oxidative stress. Furthermore, the cell viability results indicated that piceatannol could distinctly prevent the cell death of PC-12 cells induced by H_2_O_2_. However, rapamycin eliminated the protective effect of piceatannol on H_2_O_2_-induced PC-12 cells (Figure 2E). Consequently, these results illustrated that piceatannol reverted the impairment of PC-12 cell viability induced by H_2_O_2_ through downregulating autophagy.

### 3.3. Piceatannol Improved Mitochondrial Function of PC-12 Cells by Inhibiting Autophagy

**Figure 3:**
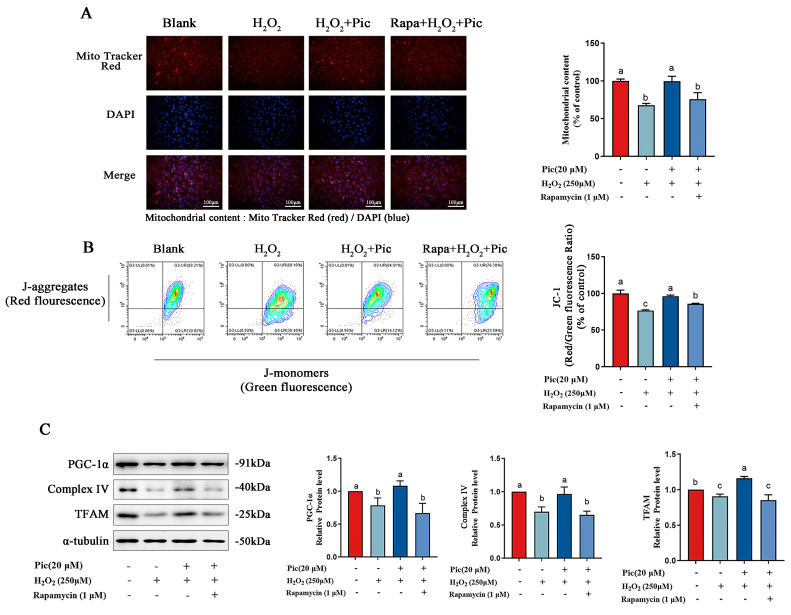
Piceatannol improved mitochondrial function of PC-12 cells by inhibiting autophagy. PC-12 cells were pretreated with piceatannol for 24 h, then treated with rapamycin for 4 h and H_2_O_2_ for another 24 h. (**A**) Mitochondrial content observed by fluorescence microscope (Magnification, ×100; Scale bar = 100 μm). (**B**) Mitochondrial membrane potential observed by flow cytometer. (**C**) The protein expression level of PGC-1α, Complex IV and TFAM measured by Western blotting assay. The data for the experiments were represented as the means ± standard deviation (SD). Results marked with the same letters were not significantly different (*p* < 0.05).

To investigate whether autophagy was involved in the regulation of mitochondrial function by piceatannol, autophagy activator rapamycin was applied in H_2_O_2_-induced PC-12 cells. The result of the fluorescence staining showed that activation of autophagy abolished the promotion of mitochondrial content by piceatannol (Figure 3A). Meanwhile, the protective effect of piceatannol on mitochondrial membrane potential was also counteracted in the presence of rapamycin (Figure 3B). In addition, mitochondrial function-related protein expression (PGC-1α, Complex IV, and TFAM) enhanced by piceatannol were significantly reduced after co-treatment with rapamycin (Figure 3C). These results demonstrated that the activator of autophagy could reverse the protection of piceatannol on mitochondrial function in PC-12 cells. 

### 3.4. Piceatannol Protected PC-12 Cells against Mitochondrial Dysfunction by Inhibiting Autophagy via SIRT3 Pathway

**Figure 4:**
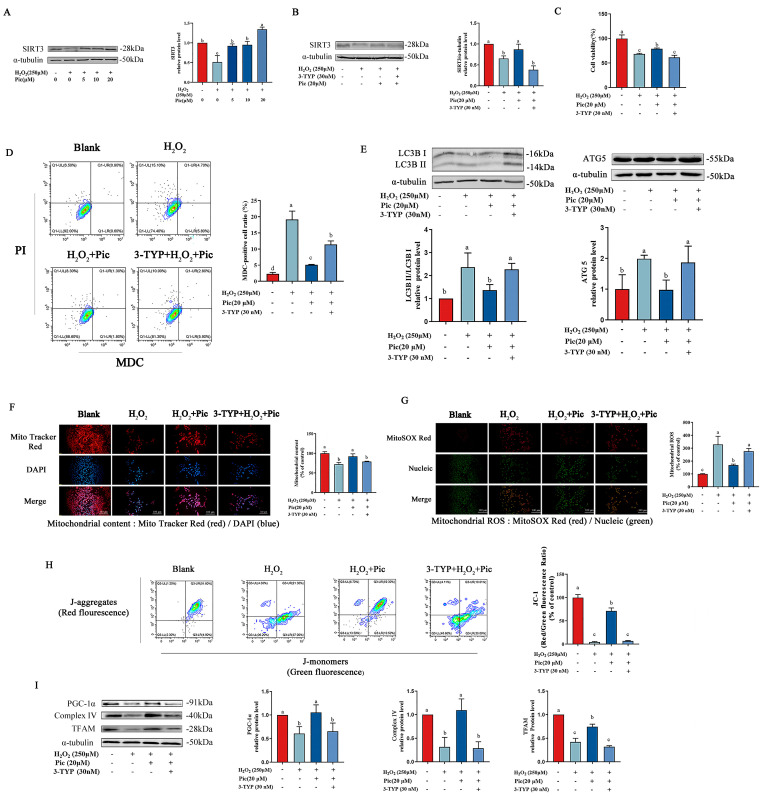
Piceatannol improved mitochondrial function of PC-12 cells by inhibiting autophagy via SIRT3 signaling pathway. (**A**) PC-12 cells were preincubated with 0, 5, 10 and 20 μM piceatannol for 24 h, followed by incubation with or without 250 μM H_2_O_2_ for another 24 h. The protein expression level of SIRT3. (**B**) PC-12 cells were pretreated with piceatannol for 24 h, then treated with 3-TYP for 4 h and H_2_O_2_ for another 24 h. The protein expression level of SIRT3 measured by Western blotting assay. (**C**) The cell viability detected by CCK8 assay. (**D**) The MDC/PI probe staining observed by flow cytometer. (**E**) The protein expression of LC3B II/I and ATG5 measured by Western blotting assay. The mitochondrial content (**F**) and mitochondrial ROS (**G**) observed by fluorescence microscope (Magnification, ×100; Scale bar = 100 μm). (**H**) Mitochondrial membrane potential measured by flow cytometer. (**I**) The protein expression level of PGC-1α, Complex IV and TFAM measured by Western blotting assay. The data for the experiments were represented as the means ± standard deviation (SD). Results marked with the same letters were not significantly different (*p* < 0.05).

As shown in Figure 4A, H_2_O_2_ exposure significantly downregulated the SIRT3 protein level compared with the control group, while pretreatment with piceatannol successfully restored the SIRT3 expression level. To investigate whether SIRT3 was involved in the protective effect of piceatannol on mitochondrial function, 3-TYP (inhibitor of SIRT3) was used to disturb SIRT3 signaling pathway in PC-12 cells. Compared with the piceatannol-pretreated group, the protein expression level of SIRT3 was significantly downregulated in PC-12 cells after co-treatment with piceatannol and 3-TYP (Figure 4B). Furthermore, inhibition of SIRT3 by 3-TYP dramatically diminished the protective effect of piceatannol (Figure 4C). Meanwhile, PC-12 cells co-treated with 3-TYP and piceatannol exhibited increased autophagosomes under oxidative stress, compared with the piceatannol treatment group (Figure 4D). 3-TYP significantly increased the protein expression level of LC3B-II/I and ATG5 which were downregulated by piceatannol (Figure 4E). 

Additionally, the protective effect of piceatannol on mitochondrial function in PC-12 cells was measured after SIRT3 inhibitor treatment. The results revealed that 3-TYP dramatically reduced mitochondrial content while elevating mitochondrial ROS levels in PC-12 cells compared to the piceatannol treatment group (Figure 4F,G). Similarly, 3-TYP markedly decreased MMP protected by piceatannol in PC-12 cells under oxidative stress (Figure 4H). In addition, piceatannol-upregulated protein (PGC-1α, Complex IV and TFAM) expression levels were also significantly reduced by 3-TYP (Figure 4I). These results illustrated that the SIRT3 inhibitor significantly inhibited the protective effect of piceatannol, suggesting the mitochondrial protective role of piceatannol in autophagy inhibition was via the SIRT3 signaling pathway.

## 4. Discussion

Mitochondria plays a crucial role in neuronal cell function which acts as a regulator of energy metabolism, while mitochondrial dysfunction leads to abnormal energy metabolism which is associated with neuronal cell death [21,28]. Moreover, mitochondrial dysfunction caused by oxidative stress is one of the factors contributing to many neurodegenerative diseases [29]. Therefore, a potential therapy for neural diseases is to maintain and reinforce mitochondrial function. Mitochondrial quality is characterized by mitochondrial biogenesis [30] and mitochondrial oxidative phosphorylation (OXPHOS) system [31]. PGC-1α regulates the production of various ROS-detoxifying enzymes and plays a key role in mitochondrial biogenesis and oxidative phosphorylation [32]. Previous studies implied that PGC-1α overexpression protected neural cells from oxidative stress-induced death, while PGC-1α deficiency affected mitochondrial structure, leading to elevated mitochondrial ROS levels and promoting cellular senescence [33,34]. Established studies showed that piceatannol promoted the transcription and activation of PGC-1α in hepatic cells and renal cells [35], and also possessed a cytoprotective effect on improving ROS-induced mitochondrial dysfunction in PC-12 cells [23]. Our findings showed that piceatannol eliminated the content of mitochondrial ROS, associated with upregulating PGC-1α expression level. In addition, mitochondrial biogenesis was regulated by TFAM, while TFAM downregulation causes mitochondrial OXPHOS deficiency, resulting in increased ROS production [36]. Additionally, TFAM overexpression significantly alleviated mtDNA oxidative damage [37]. Increasing evidence has proved that the upregulation of TFAM is a feasible intervention to alleviate neurodegenerative disease [38]. In this study, piceatannol was found to inhibit mitochondria content loss through the upregulation of TFAM. Moreover, mitochondrial respiratory chain complexes, including Complex I-V are essential for maintaining mitochondrial function in highly metabolic tissues. For instance, piceatannol was reported to reduce hepatocyte apoptosis by enhancing the activities of mitochondria Complex I, II, III, and V [21]. Additionally, piceatannol exerted a protective effect against hypoxia-induced toxicity in H9c2 cardiomyocytes by increasing the expression of the mitochondrial antioxidant enzyme [39]. Our study also showed that piceatannol enhanced the expression of mitochondria Complex IV, indicating the possible protective role of piceatannol for mitochondrial function. 

Autophagy is a process of the degradation of intracellular organelle and proteins in lysosomes that is regulated by a variety of signal molecules and pathways. Many studies have reported that autophagy disorder was involved in the development of neurodegenerative diseases [40]. In neuronal cells, the overproduction of reactive oxygen species promoted autophagy flux and resulted in cell death, while the inhibition of autophagy protected the neurons from cell death [41]. It has been reported that H_2_O_2_-induced PC-12 cell autophagy was via increasing LC3B II/LC3B I ratio and decreasing P62 expression [42,43]. LC3B is located on the membrane surfaces of pre-autophagic and autophagic vacuoles, reflecting autophagy activity to some extent [44]. ATG5 is a key molecule in the elongation phase of autophagy and knockout of ATG5 blocks autophagy [45]. In this study, H_2_O_2_ significantly promoted the autophagic protein (LC3B and ATG5) expression level in PC-12 cells, which was consistent with those reported studies. Meanwhile, piceatannol pretreatment markedly reversed the upregulation of LC3B and ATG5 and significantly reduced the intracellular autophagosome number in PC-12 cells under oxidative stress. Moreover, rapamycin markedly inhibited the protective effect of piceatannol on autophagy and cell death, suggesting that piceatannol promoted cell viability by alleviating exaggerated autophagy. Other phytochemicals were found to display similar functions. For example, berberine protected PC-12 cells from oxidative damage by inhibiting hydrogen peroxide-induced elevation of autophagy [46]. It was verified that the modulation of mitochondrial dysfunction and autophagy in PC-12 cells exhibited a protective effect on cell survival and improved redox-mediated neurological disorders [27].

SIRT3, a nicotinamide adenine dinucleotide (NAD^+^)-dependent enzyme, was reported to play a key role in the modulation of mitochondrial function and is considered a therapeutic target of neurodegeneration diseases [47]. It has been illustrated that SIRT3 activation presented the neuroprotective effect, reducing the beta-amyloid (Aβ) and Tau accumulation which contributed to oxidative neurotoxicity in the brain tissues of AD animals [48]. Meanwhile, Ying et al. found that SIRT3 showed neuroprotective effects by eliminating mitochondrial ROS [49]. However, SIRT3 deficiency exacerbated neuronal degeneration through the progressive accumulation of ROS caused by abnormal autophagy, cell apoptosis and mitochondrial dysfunction [50,51]. Piceatannol was proven to enhance the expression level of sirtuins (SIRT) including SIRT1, SIRT3, SIRT6 and the downstream target of SIRT3, PGC-1α, which improved inflammatory effect and insulin resistance in high-fat-diet fed rats [52]. In a previous study, piceatannol protected neurons against oxidative damage via upregulating anti-oxidative enzyme expression and modulating the protein expression of SIRT3 as well as its downstream FOXO3a signaling in PC-12 cells [23]. Here, we found that 3-TYP dramatically reversed the protective effect of piceatannol on mitochondrial function via downregulating SIRT3 expression. Furthermore, 3-TYP dramatically enhanced mitochondrial ROS production and aggravated mitochondrial content loss in PC-12 cells with piceatannol treatment. Meanwhile, our findings showed that 3-TYP downregulated the expression of mitochondria-related proteins (TFAM, PGC-1α and Complex IV) in PC-12 cells treated with piceatannol, revealing that the protection by piceatannol against oxidative stress was via activating SIRT3 signaling pathway.

Furthermore, the results of this study indicated that SIRT3 was involved in the neuroprotective effects of piceatannol on mitochondria-mediated autophagy. In line with this, Rodriguez-Enriquez et al. found that mitochondrial ROS were primarily generated by damaged mitochondria, typically the ones with abnormal mitochondrial membrane potential which acted as an indicator of mitochondria-mediated autophagy [53]. For neuronal cells, SIRT3 exhibited different regulatory effects on autophagy depending on the inducing conditions. For instance, the plant-derived bioactive component, scopoletin, was reported to exert neuroprotective effects by inhibiting the autophagy of SH-SY5Y cells via SIRT3 signaling [15]. Resveratrol increased autophagy by promoting SIRT3 expression to protect neuronal HT22 cells under tunicamycin-induced endoplasmic reticulum stress [16]. In our study, the inhibitory effect of piceatannol on the autophagy of PC-12 cells was alleviated when the SIRT3 signaling was inhibited. These findings indicated that piceatannol could mitigate autophagy by improving mitochondrial function mediated by SIRT3 signaling.

## 5. Conclusions

In conclusion, the cytoprotection of piceatannol against oxidative damage was verified in PC-12 cells. The findings in this study indicated that piceatannol could significantly inhibit the cell death induced by H_2_O_2_ by ameliorating the overactivated autophagy. Moreover, the activation of the SIRT3 signaling pathway by piceatannol significantly reduced H_2_O_2_-induced mitochondrial dysfunction and autophagy. These findings suggest that piceatannol holds the promise of a potential bioactive component for neuron protection against oxidative stress, contributing to the development of piceatannol-based products to prevent neurodegenerative disorders. 

## Data Availability

The data that support the findings of this study are available from the corresponding author upon reasonable request.

## Abbreviations

Aβ, beta amyloid; AD, Alzheimer’s disease; ATG5, autophagy-related protein 5; ATP, adenosine triphosphate; AVOs, acidic vesicular organelles; FOXO3a, Forkhead box O3; LC3B, light chain 3B; MDC, monodansylcadavrine; MMP, mitochondrial membrane potential; NAD, nicotinamide adenine dinucleotide; OXPHOS, oxidative phosphorylation; PC-12, Pheochromocytoma-12; PD, Parkinson’s disease; PGC-1α, peroxisome-proliferator-activated receptor-γ coactivator-1α; PI, propidium iodide; Pic, piceatannol; Rapa, rapamycin; ROS, reactive oxygen species; SIRT3, sirtuin 3; TFAM, mitochondrial transcription factor A; 3-TYP, 3-(1H-1,2,3-Triazol-4-yl) pyridine.
